# Identification of a prognostic metabolic gene signature in diffuse large B‐cell lymphoma

**DOI:** 10.1111/jcmm.16720

**Published:** 2021-06-14

**Authors:** Huizhong Wang, Ruonan Shao, Wenjian Liu, Hailin Tang, Yue Lu

**Affiliations:** ^1^ Sun Yat‐sen University Cancer Center Guangzhou China; ^2^ State Key Laboratory of Oncology in South China Guangzhou China; ^3^ Collaborative Innovation Center for Cancer Medicine Guangzhou China

**Keywords:** clinical prognostic model, diffuse large B‐cell lymphoma, gene signature, metabolism

## Abstract

Diffuse large B‐cell lymphoma (DLBCL) is a clinically diverse disease. Given the numerous genetic mutations and variations associated with it, a prognostic gene signature that can be related to the overall survival (OS) is a clinical implication. We used the mRNA expression profiles and clinicopathological data of patients with DLBCL from the Gene Expression Omnibus (GEO) database to identify a metabolism‐related gene signature. Using LASSO regression analysis, a novel 13‐metabolic gene signature was identified to evaluate prognosis. The information gathered was used to construct the nomogram model to improve risk stratification and quantify risk factors for individual patients. We performed gene set enrichment analysis to identify the enriched signalling axes to further understand the underlying biological pathways. The receiver operating characteristic (ROC) curve revealed a satisfactory performance in the training cohorts. The model also showed clinical benefit when compared to the standard prognostic factors (*P* < .05) in validation cohorts. This study aimed to combine metabolic dysregulation with clinical features of patients with DLBCL to generate a prognostic model that might not only indicate the value of the metabolic microenvironment for prognostic stratification but also improve the decision‐making during individual therapy.

## 1 INTRODUCTION

Diffuse large B‐cell lymphoma (DLBCL) is a highly prominent class of non‐Hodgkin lymphoma (NHL), accounting for 30%‐40*%* of all NHL cases. It has been reported to be highly heterogeneous in epidemiological studies.^1^, ^2^ Though anti‐CD20 (rituximab) immunotherapy has been the standard treatment regimen with satisfactory survival rates for decades,^3^ it was only effective on a small portion of patients. Approximately 40% of patients with DLBCL still had poor prognoses and often suffered from an early relapse after the treatment,^4^ emphasizing the need for risk stratification to provide accurate and effective therapy. The International Prognostic Index (IPI),^5^ involving conventional clinical and pathological parameters, is widely used for prognosis of patients with DLBCL. However, IPI excludes cytogenetic, genomic and molecular mechanistic characteristics that may have a strong impact on the prognosis. The cell‐of‐origin (COO) categorization divides patients with DLBCL into subcategories, based on their transcriptional profile: germinal centre B cells (GCB), activated B cell (ABC) and unclassified.^6^ Moreover, it has been emphasized that the genomic prognostic characteristics and their interaction with other genetic and clinical factors can play a considerable role in guiding the design and interpretation of future clinical trials.^7^ Therefore, it is crucial to better understand the pathogenesis of DLBCL and identify the effective therapeutic targets, and novel prognostic biomarkers with additional factors for risk stratification.

Metabolism is an indispensable feature of tumour biology. As such, metabolism and its corresponding biological effects have become an important feature in the comprehension of cancer aetiology, tumour progression and underlying pathophysiological changes.^8^ According to multiple reports, cellular metabolism is intricately involved with numerous signal transduction pathways and plays an essential role in several disease states.^9^, ^10^ Consequently, blocking the implicated metabolic pathways can sometimes prove to be an effective treatment strategy.^11^ Several studies have found associations between the pathogenesis, metabolism and management of DLBCL. The PI3K/AKT/mTORC1 axis, for instance, has been shown to activate cell pro‐survival factors and reprogram B‐cell lymphoma fatty acid metabolism, glycolysis and tricarboxylic acid cycle to activate lymphoma cell proliferation, invasion and migration.^12^, ^13^

In particular, a model based on multiple metabolic genes should be superior for predicting prognosis, to a single gene, as metabolism is a polygenic process. With advancements in high‐throughput genome sequencing technology, gene signatures, in combination with classical clinicopathological characteristics, can be used for the diagnosis and prognosis of lymphoma. However, no studies have attempted to describe the metabolic characteristics of DLBCL for prognosis. Therefore, we designed a metabolic gene‐based prognostic model, based on data from the Gene Expression Omnibus (GEO) database, to identify a useful metabolic gene signature for DLBCL. We also generated a nomogram from the metabolic gene signature and clinical manifestation to estimate patient overall survival (OS). Our analysis will enhance the personalized therapeutic strategies for patients with DLBCL.

## 2 MATERIALS AND METHODS

### 2.1 Data collection

The mRNA profiles and clinical information of the three study populations were obtained from the GEO databases: GSE10846,^14^ GSE23501 ^15^ and GSE4732.^16^ Simultaneously, comprehensive patient medical records, including age, sex, DLBCL stage, LDH levels, ECOG score, IPI score, number of extranodal sites and survival information, were also retrieved from the GEO data set. Groupings of metabolic gene sets were downloaded from the GSEA c2.cp.kegg.v7.0.symbols Molecular Signatures Database (MSigDB). To select genes for subsequent analysis, Perl scripts were employed to identify metabolic genes in the intersection of the GEO cohorts and the MSigDB gene sets.

### 2.2 Prognostic metabolic risk model

The GSE10846 data set was used as the training cohort. The most appropriate weighting coefficient for the metabolic genes was determined by the least absolute shrinkage and selection operator (LASSO) Cox regression analysis. Next, 1,000‐fold cross‐validation was used to penalize the maximum likelihood estimator. Lastly, a general formula established based on the training cohort was used to assess the metabolic risk score, and the patients were assigned to one of the two groups: high risk (HR) or low risk (LR). Forward stepwise univariate and multivariate Cox regression analyses were used to assess the independence of the risk score for prognosis prediction in the training and validation cohorts. The two‐sided *P* < .05 was determined as being statistically significant.

### 2.3 Gene set enrichment analysis

The c2.cp.kegg.v7.0.symbols gene set, downloaded from MSigDB, was used to identify relevant enriched biological pathways with the GSEA v4.0.2 software (http://software.broadinstitute.org/gsea/login.jsp). This also included a validation queue for rich pathway analysis. *P* < .05 was defined as statistically significant. The analysis of the interaction between model‐related metabolic proteins and related proteins was carried out using Gene Cloud Biotechnology Information (GCBI) and Cytoscape 3.7.2.

### 2.4 Bioinformatics and statistical analysis

The receiver operating characteristic (ROC) curve was drawn over time, and the prognostic value of candidate factors was evaluated and compared by calculating the area under the ROC curve (AUC). We defined OS as the primary outcome, from the date of admission into study till death. The "survival" software package was employed to draw the Kaplan‐Meier curves, and the log‐rank test was used for comparison. Using univariate and multivariate Cox analysis, the influence of clinical and genetic information on prognosis was discussed. Categorical variables were compared using chi‐square test or Fisher's exact test. A nomogram was generated to illustrate the metabolic characteristics for visualization and integration of OS, based on the calibration evaluation data. Statistical significance (*P* < .05) was measured using R software (version 3.6.0) and SPSS version 24.0 (SPSS, Inc) software.

## 3 RESULTS

### 3.1 Patient characteristics

**FIGURE 1:**
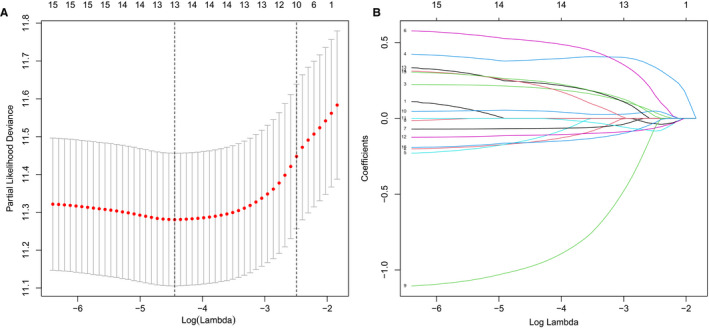
Construction of the metabolic model for DLBCL. A, 1,000‐fold cross‐validation for variable selection in the LASSO regression via min criteria. B, LASSO coefficients of metabolism‐related genes. Each curve represents a metabolic gene

This analysis included 579 patients with available survival data in 3 cohorts, obtained from the GEO database. GSE10846 (n = 331) was regarded as the training cohort and was the basis for building the prognostic metabolic model. Patients in the GSE4732 (n = 174) and GSE23501 (n = 60) cohorts served as the external validation cohorts. A summary of our data collection strategy can be found in Figure 1; patients with incomplete clinical features were not included. Patients from the GSE10846 cohorts were aged 14‐92 years (median age, 58.85 years), whereas those in the GSE4732 and GSE23501 cohorts had a median age of 62.21 (range: 14‐92) years and 63.78 (range: 19‐92) years, respectively. A comprehensive summary of patient characteristics in the three cohorts is summarized in Table 1.

**TABLE 1:** The detailed characteristics of patients and correlation between clinicopathological features and metabolic risk level in training cohort and two external validation cohorts in DLBCL

Characteristics	Training cohort		*P*‐value	Validating cohort 1		*P*‐value	Validating cohort 2		*P*‐value
Risk	High risk	Low risk		High risk	Low risk		High risk	Low risk	
Patient	168	163		99	75		15	45	
Gender									
Male	85	91	.062	54	44	.645	12	31	.52
Female	71	69		45	31		3	14	
Age									
≤60 y	69	90	.01	36	37	.091	8	17	.369
>60 y	99	73		63	38		7	28	
Stage			.158	4		.018			.761
Ⅰ/Ⅱ	71	86		46	49		6	21	
Ⅲ/Ⅳ	96	76		49	26		8	21	
ECOG									
<2	121	131	.075	68	60	.095	‐	‐	‐
≥2	47	32		27	15		‐	‐	
Subtype									
GCB	33	117	<.01	20	57	<.01	2	38	<.01
Unclassified/ABC	135	46		79	18		13	7	
Number of extranodal sites									
<2	157	147	.181	70	63	.122	‐	‐	‐
≥2	11	13		20	8		‐	‐	
LDH									
<	74	91	.025	46	48	.058	‐	‐	‐
≥	93	68		49	26		‐	‐	
IPI									
0,1	57	83	.006	28	41	.005	‐	‐	‐
2	57	38		31	19		‐	‐	
3	31	28		21	10		‐	‐	
4,5	23	14		19	5		‐	‐	
Treatment regimens									
CHOP	108	50	<.01	‐	‐	‐	‐	‐	‐
RCHOP	60	113		‐	‐		‐	‐	
B‐symptom									
Yes	‐	‐	‐	‐	‐	‐	5	12	.739
No	‐	‐		‐	‐		9	30	

Abbreviations: ABC, activated B cell; ECOG, Eastern Cooperative Oncology Group; GCB, germinal centre B cell; IPI, International Prognostic Index.

### 3.2 A prognostic metabolic model

The prognostic metabolic signature was constructed using GSE10846 by the LASSO Cox regression analysis (Figure 1A). From the metabolic gene set, a subset of 13 genes and their weighting coefficients were identified by the LASSO Cox method. The expression levels of the genes and the weighting coefficients were then employed to calculate the risk score of individual patients using the formula: risk score = −0.155 × *BPNT1* levels+0.203 × *CTH* levels +0.385 × *DCTD* levels −0.135 × *DNMT1* levels +0.511 × *GLO1* levels – 0.066 × *ITPKB* levels – 0.971 × *LDHA* levels +0.050 × *MPI* levels ‐ 0.111 × *PDE9A* levels +0.236 × *POLR1C* levels +0.224 × *POLR3A* levels +0.248 × *POLR3H* levels – 0.158 × *PTGDS* levels. The Survminer R package was used to identify the optimal risk cut‐off score.

Next, with the risk scores calculated, we chose the time‐related ROC and Kaplan‐Meier curves to perform time‐dependent evaluation.

### 3.3 Metabolic risk score evaluation

**FIGURE 2:**
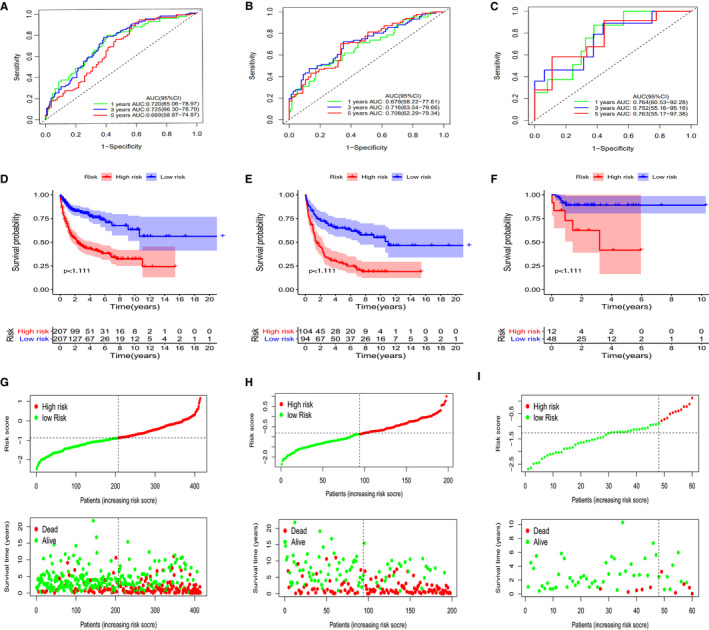
Time‐dependent ROC analysis, survival outcome analysis and Kaplan‐Meier analysis and risk score analysis for the 13‐gene signature in DLBCL. A–C, Time‐dependent ROC analysis for 1‐, 3‐ and 5‐year overall survival (OS) of prognostic model in training cohort and the validation cohorts of GSE4732 and GSE23501. D–F, Kaplan‐Meier curve of the prognostic model in the training cohort the validation cohorts of GSE4732 and GSE23501. G–I, Kaplan‐Meier curve of the prognostic model in the three chorts Mentioned above. Risk score analysis of the 13‐gene signature in the training cohort and the validation cohorts of GSE4732 and GSE23501

The HR group had significantly lower OS rates than did the LR groups (*P* < .01; Figure 2). The sensitivity and specificity of the metabolic risk model were evaluated using time‐dependent ROC curves. The AUCs for 1‐, 3‐ and 5‐year OS were 0.720 (95% CI: 65.06‐78.97), 0.725 (95% CI: 66.30‐78.79) and 0.699 (95% CI: 58.87‐74.87), respectively, within the cohort GSE10846 (Figure 2A). Furthermore, in the remaining two verification cohorts, risk scores were calculated using the same formula mentioned above. The GSE4732 cohort AUCs for 1‐, 3‐ and 5‐year OS were 0.679 (95% CI: 58.22‐77.61), 0.716 (95% CI: 63.54‐79.66) and 0.708 (95% CI: 62.29‐79.34), respectively (Figure 2B). In the GSE23501 cohort, the AUCs were 0.764 (95% CI: 60.53‐92.28), 0.752(95% CI:55.16‐95.16) and 0.763(95% CI: 55.17‐97.38), respectively (Figure 2C). Based on these data, the 13‐gene signature was effective for estimating OS in patients with DLBCL. The patients were then assigned into one of two categories, HR or LR, based on the median risk score. As depicted in Figure 2D‐I, in all cohorts, the HR groups had a worse prognosis and shorter OS times relative to patients in the LR group (*P* < .05). Thus, we demonstrated that the metabolic prognostic panel was superior for estimating the prognosis of DLBCL over standard IPI scoring.

### 3.4 Metabolic gene signature as an independent prognostic tool

**FIGURE 3:**
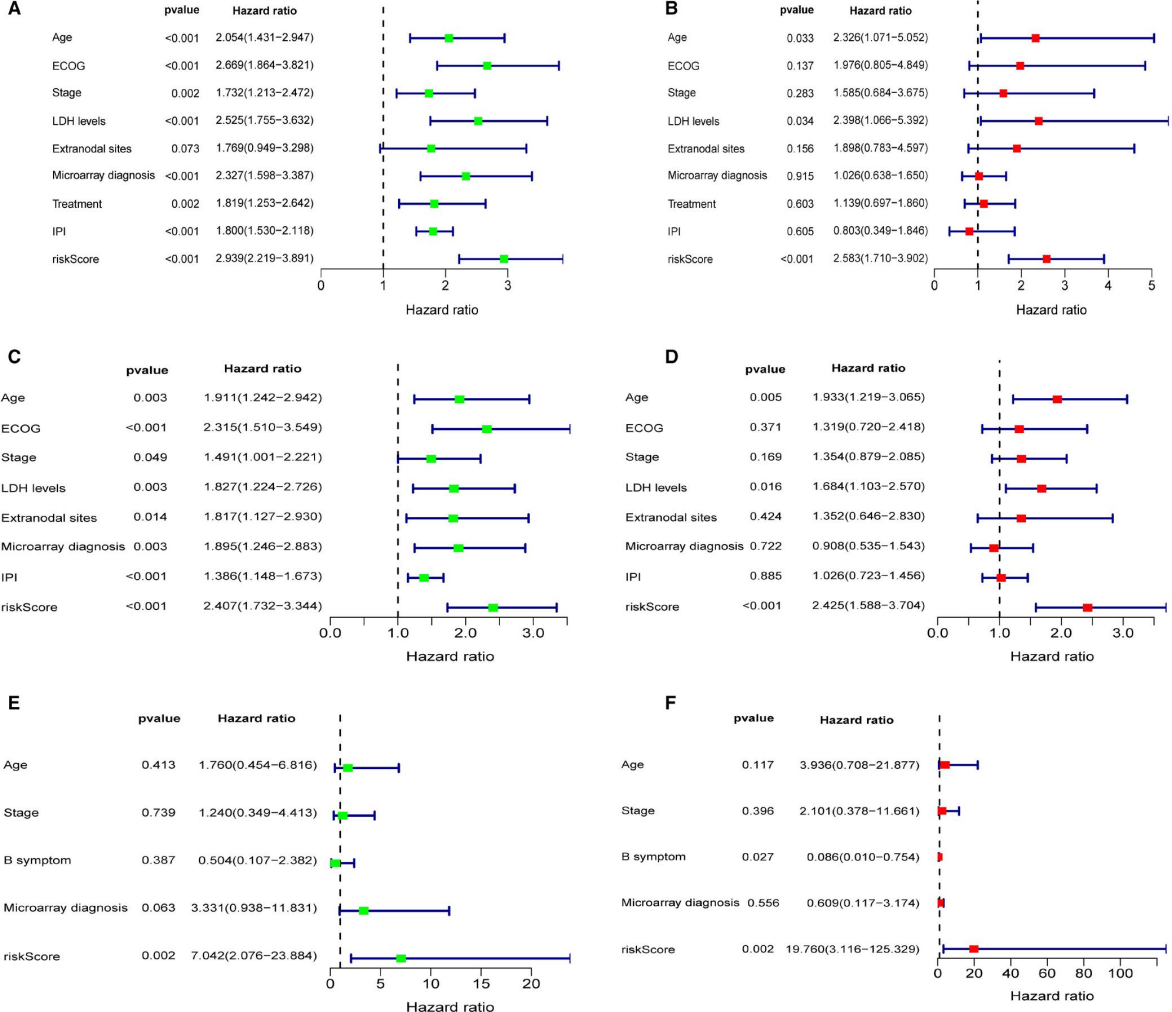
Forrest plot of the univariate and multivariate Cox regression analyses. Forrest plot of the univariate and multivariate Cox regression analyses in the training cohort (A, B). Forrest plot of the univariate and multivariate Cox regression analyses in the validating cohort of GSE4732 (C, D) and the validation cohorts of GSE23501 (E, F)

Using the univariate Cox analysis for risk score and clinical characteristics, we demonstrated a correlation between OS of patients with DLBCL and age, IPI, ECOG, stage, LDH levels, number of extranodal sites, subtype and the risk score in the training cohort (Figure 3A). A subsequent multivariate analysis revealed that the risk score can be effectively used as a stand‐alone estimator of OS in patients with DLBCL, with a hazard ratio of 2.583 (95% CI: 1.710‐3.902) in the cohort GSE10846 (Figure 3B), 2.425 (95%CI: 1.588‐3.704) in the cohort of GSE4732 (Figure 3D) and 19.760 (3.116‐125.329) in the cohort of GSE23501 (Figure 3F), after adjustment for clinical covariates in the external validating cohort.

### 3.5 Clinicopathological features in the context of risk levels

**FIGURE 4:**
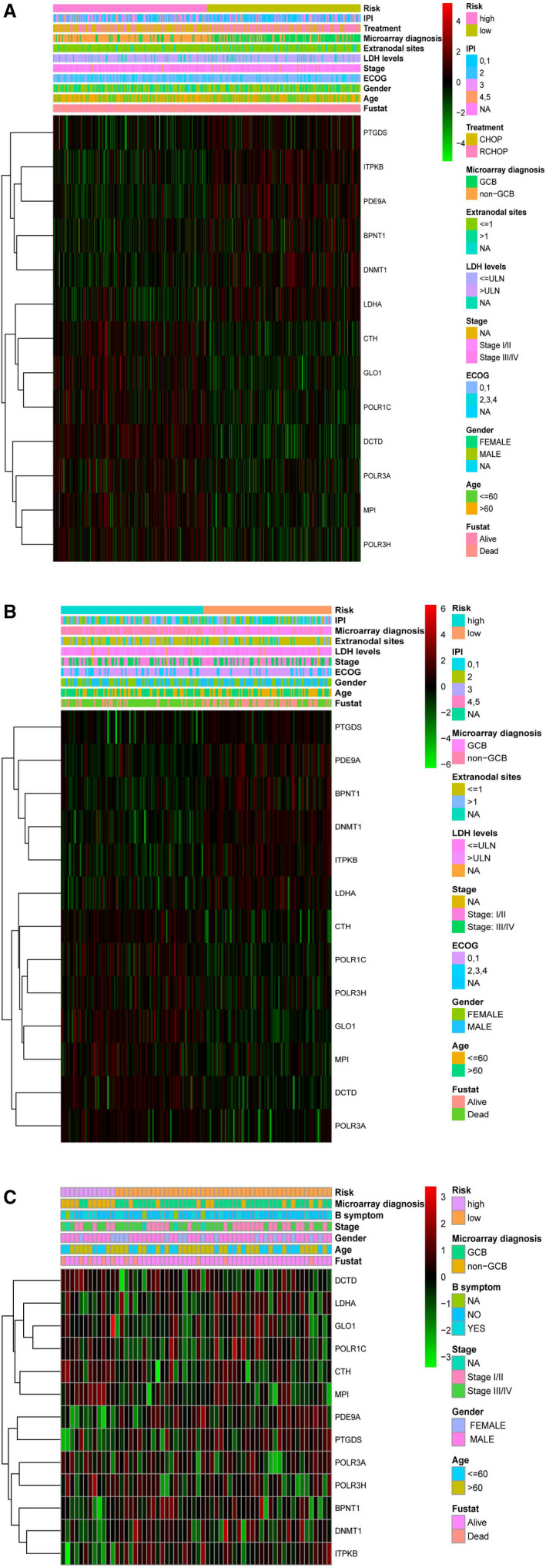
Heatmap of the 13‐gene signature and clinicopathological characteristics in different metabolic risk levels for training cohort (A) and validation cohorts of GSE4732 (B) and GSE23501 (C). Each column showing gene expression or clinicopathological state represents a sample, and each row represents one characteristic or gene in the model. The expression levels of the 13 genes are shown in different colours. Blue and yellow indicate low‐ and high‐risk levels. ECOG, Eastern Cooperative Oncology Group; IPI, International Prognostic Index; ABC, activated B cell; GCB, germinal centre B cell

The age, subtype, LDH levels, IPI score and treatment regimens in the HR training cohort and the stage, subtype and IPI score in the HR GSE4732 cohort are summarized in Figure 4A,B and Table 1. We did not observe a statistical difference in the clinical features of HR and LR GSE23501 cohort, except for subtype, owing to a small study population (Figure 4C and Table 1). Lastly, associations between the clinicopathological features and the metabolic transcript profiles of all patients with DLBCL studied are shown in Figure 4.

### 3.6 GSEA and external validation

**FIGURE 5:**
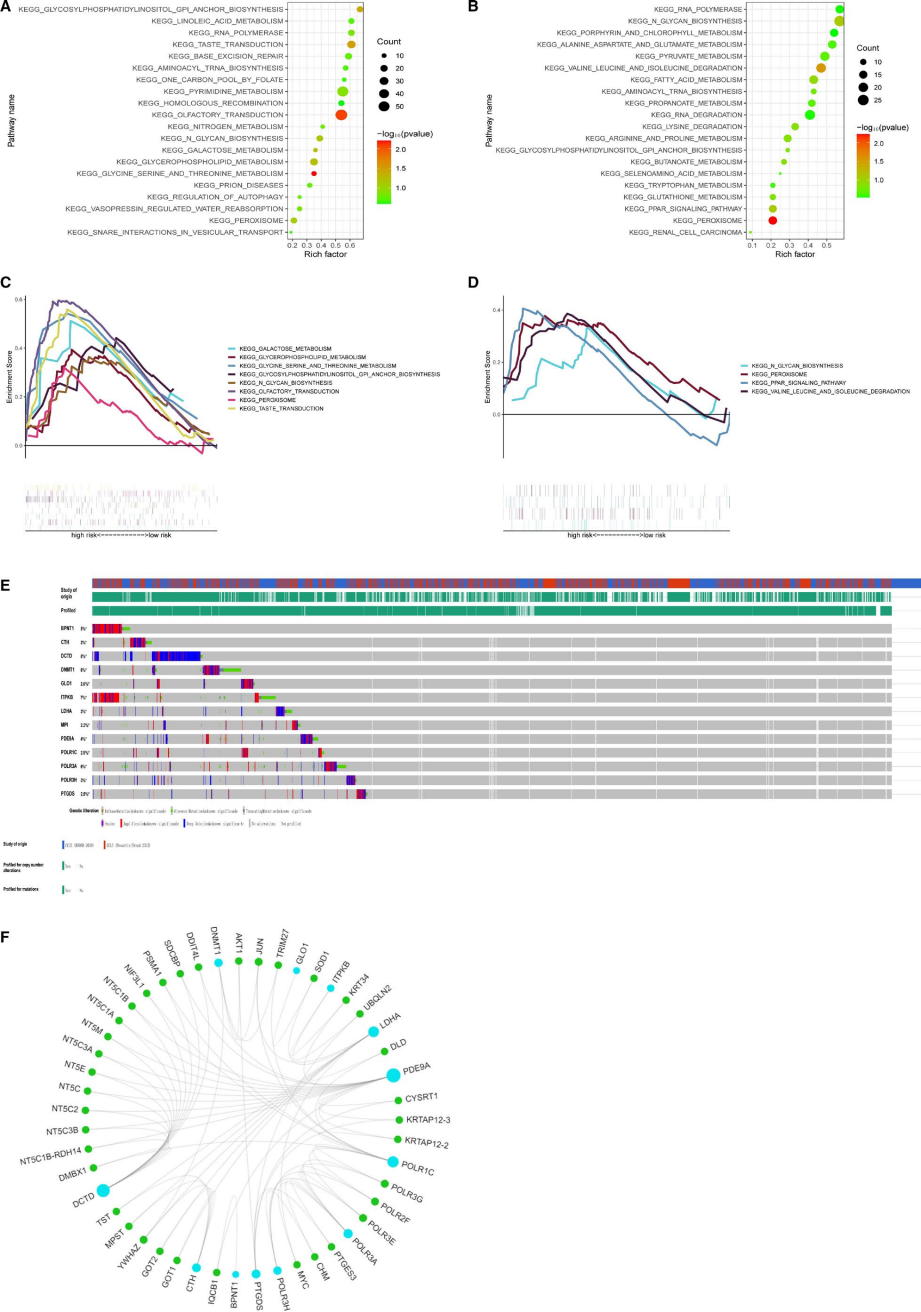
Significantly enriched KEGG pathways in three cohorts by GSEA. Genetic alterations of the 13 genes based on GSEA. A, B, Top 20 representative KEGG pathways in high‐risk patients in the training cohort and the cohort of GSE23501 (*P* <.05). C, D, Representative metabolic pathways in high‐risk patients in the training cohort and the cohort of GSE23501. E, Genetic alterations of the 13‐gene panel in CCLE, obtained from the cBioportal for cancer genomics. F, The protein‐protein interactions between the metabolic model‐related proteins and the other proteins. The model‐related proteins are shown in blue circles, and the size of which is determined by the number of interacting proteins. MPI has no known interactions with other proteins

GSEA was performed to identify the markedly enriched KEGG pathways within the three cohorts. We found that a large portion of metabolic signalling was enriched in the HR group, as opposed to LR (Figure 5A–D). Among these were the galactose, glycerophospholipid, fatty acid and glycine serine and threonine metabolic pathways, as well as N‐glycan biosynthesis, PPAR signalling, GPI‐anchor biosynthesis pathway, RNA degradation and peroxisome regulatory pathways (Figure 5A–D).

We then explored the mutant variants of the metabolic gene panel in the Cancer Cell Line Encyclopedia database (CCLE, https://portals.broadinstitute.org/ccle) via the cBioPortal for Cancer Genomics (http://www.cbioportal.org/). ^17^ Among the 579 patients analysed, gene amplification was the most apparent form of dysregulation in the patients with DLBCL (Figure 5E). *BPNT1*, *DCTD*, *DNMT1*, *ITPKB* and *POLR3A* genes were commonly altered. Further, abnormal metabolic gene expression has been verified in lymphoma cell lines, providing evidence that the genetic changes represent alterations in the underlying metabolisms of lymphoma cells.

We identified 48 DLBCL‐related proteins from the GCBI database. Among them, 12 were related to our metabolic model, with the exception of the MPI. Using the PPI network, we discovered that the *DCTD* and *PDE9A* proteins physically interacted with the 5'‐nucleotidase type 3 family of proteins (ie *NT5C1B, NT5C1A, NT5 M, NT5C3A, NT5E, NT5C, NT5C2, NT5C3B,* Figure 5F). Based on these data, genetic alterations that promote metabolic changes likely contribute to DLBCL

### 3.7 Comparing prognostic factors and merged risk scores

**FIGURE 6:**
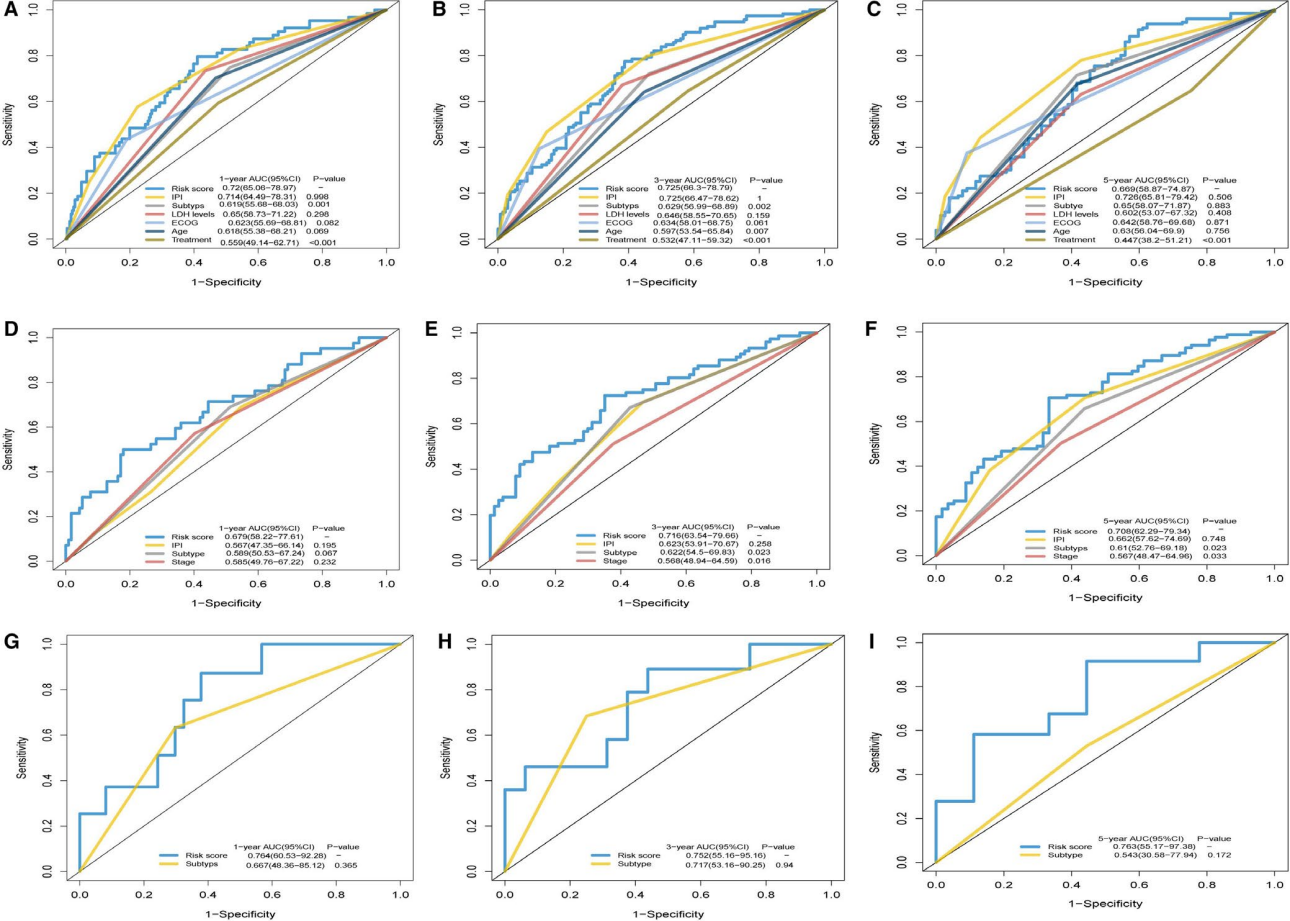
Time‐dependent receiver operating characteristic (ROC) analysis of 1‐, 3‐ and 5‐year overall survival (OS) of metabolic risk model compared with other potential prognostic factors. A‐I, Time‐dependent ROC analysis for 1‐, 3‐ and 5‐year OS of metabolic risk model in the training cohort and the validating cohorts. A, B and C display GSE10846; D, E and F display GSE4732; G, H and I display GSE23501. ECOG, Eastern Cooperative Oncology Group; IPI, International Prognostic Index

The sensitivity and specificity of metabolic features were compared with those of other potential prognostic variables using the AUCs of the ROC curves. The AUCs for 1‐, 3‐ and 5‐year OS were 0.720 (95% CI: 65.06‐78.97), 0.725(95% CI: 66.30‐78.79) and 0.699 (95% CI: 58.87‐74.87), respectively, in the training cohort. The AUC was remarkably high for the risk scores calculated using the metabolic gene‐based model compared with those from other factors such as stage, IPI, ECOG, LDH level, number of the extranodal sites and subtype (all *P* < .05, Figure 6A‐C). Furthermore, the metabolic gene‐based model produced AUCs for 1‐, 3‐ and 5‐year OS values that were higher than that for standard IPI scores, but were not significant for the GSE4732 and GSE23501 cohorts (Figure 6D‐I).

### 3.8 Validation of a predictive nomogram

**FIGURE 7:**
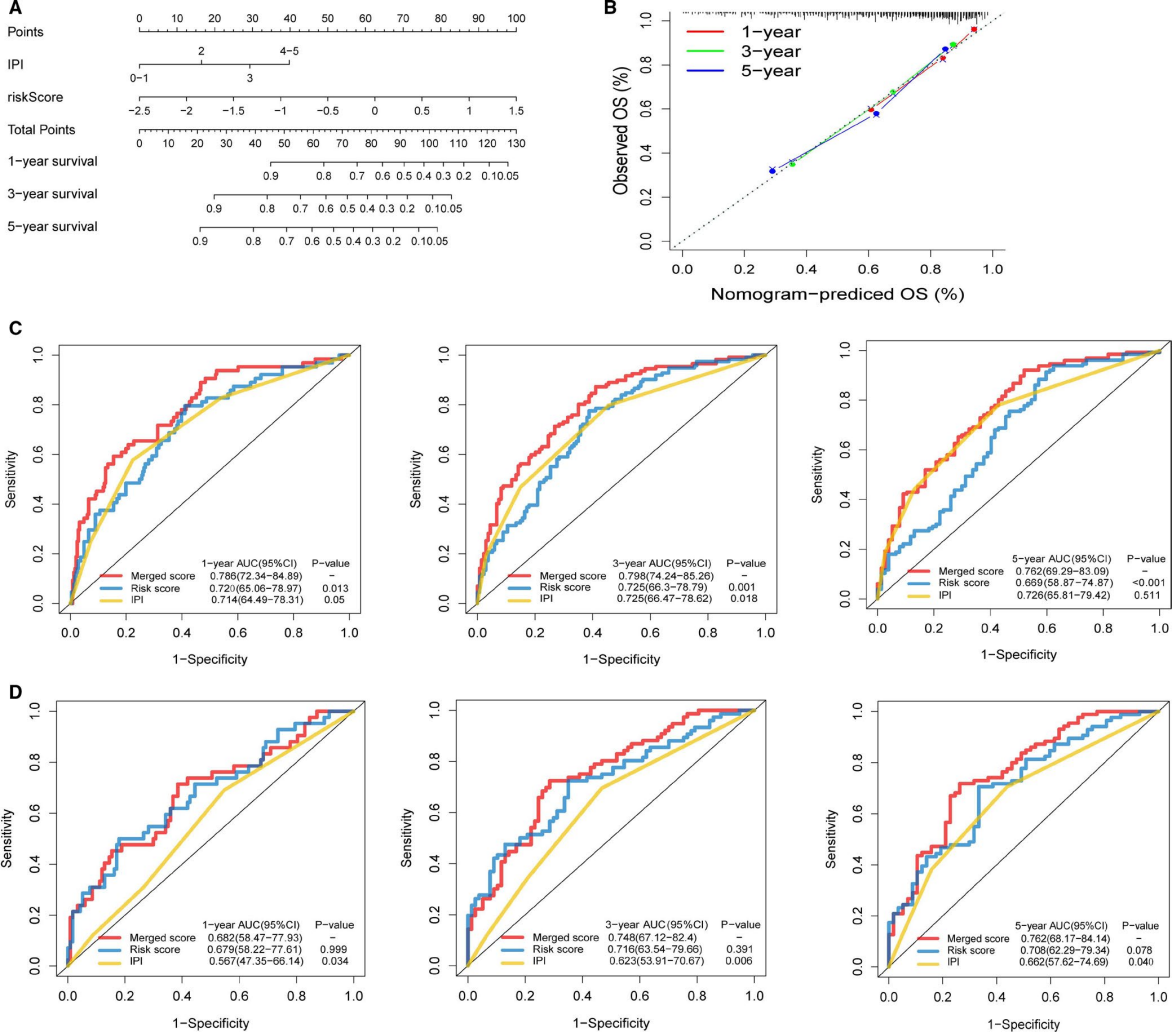
Building and validation of the nomogram to predict the overall survival of patients combining the training cohort and validation cohorts. A, Nomogram plot was built based on age, IPI score, metabolic risk score and total points combining the training cohort and validation cohorts. B, Calibration plot of the nomogram. C, D, Time‐dependent receiver operating characteristic (ROC) curves of nomograms were compared based on 1‐, 3‐ and 5‐year OS of the training cohort and the cohort of GSE4732

Next, we constructed a nomogram to estimate 1‐, 3‐ and 5‐year OS for all patients in this study, using three independent prognostic factors: IPI score, risk score and the AUC merge score (Figure 7A). According to the calibration plot, our designed nomogram correctly estimated 1‐ and 3‐year OS (Figure 7B). The AUCs of the ROC curves for 1‐, 3‐ and 5‐year OS generated using the merged score were 0.786 (95% CI: 72.34‐84.89), 0.798 (95% CI: 74.24‐85.26) and 0.762 (95% CI: 69.29‐83.09), respectively, higher than the standard IPI scores and genetic risk category in the training cohort (Figure 7C). Similarly, the AUCs for 1‐, 3‐ and 5‐year OS generated using the combined risk score for the GSE4732 cohort were higher than the standard IPI scores (Figure 7D). Based on these data, nomograms incorporating combined risk scores from metabolic gene signatures and other prognostic factors are superior to single factor–based prediction models. Though our nomogram was better at estimating short‐term survival (1‐3 years) than long‐term survival (≥5 years), it may provide crucial information for DLBCL diagnosis and treatment (Figure 7C).

## 4 DISCUSSION

Multiple researchers have demonstrated the contribution of metabolic pathways in the malignancy of lymphomas.^18^, ^19^ This led to new approaches for lymphoma diagnosis, characterization and treatment. However, there are currently no prognostic tools that include metabolic factors. Therefore, we generated a metabolic gene panel, using DLBCL‐related metabolic pathways to aid in the prognosis estimation of DLBCL.

In this study, data from the training cohort were used to generate a unique 13‐gene metabolic prognostic model, which was externally validated using two separate cohorts. The model can be employed to assign patients with DLBCL into one of two risk groups: HR or LR. We demonstrated that the metabolic gene expression model can be reliably used to predict prognosis of DLBCL, especially in terms of short‐term. Moreover, our metabolic gene panel exhibited enhanced prognostic value relative to the standard IPI score.

Most of the 13 genes, in our study, have previously been reported to contribute to cancer. *PTGDS* was reported to serve important roles in lipids and lipoprotein metabolism. Consistent with our analysis, low *PTGDS* expression was shown to be related to poor prognosis in patients with DLBCL.^19^, ^20^ *ITPKB*, implicated in actin remodelling, was previously shown to control haematopoietic stem cell homeostasis through AKT and mTOR signalling.^21^ Similarly, *DNMT1* was reported to be involved in cysteine and methionine metabolism and linked to DNA replication in DLBCL cells.^22^ Additionally, *DNMT1* has been shown to be ubiquitously expressed in primary DLBCL cells, where it increased cell proliferative and was predictive of OS in patients with DLBCL.^23^ Likewise, the *CTH* gene has also been reported to be involved in cysteine and methionine metabolism and was found to promote progression and metastasis of prostate cancer^24^ and bladder cancer.^25^ Owing to the essential role of *LDHA* in glucose metabolism, it has been implicated in tumour maintenance, alteration in tumour microenvironment and promotion of tumour growth and metastasis.^26^, ^27^ Taken together, our new metabolic gene panel suggests strong metabolic dysregulation in DLBCL and may, therefore, provide additional targets that can be used to develop effective therapy against the disease. However, further investigations into the metabolic genes and DLBCL are necessary.

Our GSEA data revealed numerous significantly enriched metabolic pathways, especially in the HR patient group, providing further confirmation of the importance of targeting metabolic genes in DLBCL therapy. It is known that an increase in the number of genome copies, among other mutations, may reduce the expression of a gene ^28^ and approximately ≥12% of human genetic mutations can be attributed to changes in copy number.^29^ These metabolic characteristics can be predicted to some extent and may hold therapeutic value. However, these findings remain to be validated in a large population randomized trial.

While our metabolic gene panel has clinical significance, certain limitations of this study must be considered. The estimation power of our risk‐scoring model was higher than that of the standard IPI score. However, the clinical features extracted from the GEO databases sometimes included limited or incomplete data. As such, data from these databases were not used in this study. Second, further investigation is warranted to determine the importance of these metabolic factors in the pathogenesis of DLBCL.

## 5 CONCLUSION

We generated a metabolic gene‐based model to predict the prognosis of DLBCL. The fact that our model, based only on genes involved in metabolism, had a high accuracy in predicting survival, reflects the disorder in the metabolic networks in the cancer cells and can potentially be used as biomarkers for the diagnosis and prognosis of DLBCL. Furthermore, this gene signature, whose prognostic performance can be used in clinical practice and functional experiments, should be further investigated to ensure its true significance in personalized therapeutic strategies.

## CONFLICT OF INTEREST

The authors declare no conflicts of interest in this work.

## AUTHOR CONTRIBUTION

**Huizhong Wang:** Conceptualization (equal); Data curation (equal); Formal analysis (equal); Methodology (equal); Resources (equal); Software (equal); Writing‐original draft (equal). **Ruonan Shao:** Conceptualization (equal); Data curation (equal); Formal analysis (equal); Methodology (equal); Resources (equal); Software (equal). **Wenjian Liu:** Formal analysis (equal); Methodology (equal); Resources (equal); Software (equal); Validation (equal); Writing‐original draft (equal). **Hailing Tang:** Project administration (equal); Supervision (equal); Writing‐review & editing (equal). **Yue Lu:** Funding acquisition (lead); Project administration (equal); Supervision (equal); Writing‐review & editing (equal).
